# Defined Tau Phosphospecies Differentially Inhibit Fast Axonal Transport Through Activation of Two Independent Signaling Pathways

**DOI:** 10.3389/fnmol.2020.610037

**Published:** 2021-01-25

**Authors:** Sarah L. Morris, Ming-Ying Tsai, Sarah Aloe, Karin Bechberger, Svenja König, Gerardo Morfini, Scott T. Brady

**Affiliations:** ^1^Department of Anatomy and Cell Biology, University of Illinois at Chicago, Chicago, IL, United States; ^2^Marine Biological Laboratory, Woods Hole, MA, United States

**Keywords:** tau phosphorylation, fast axonal transport, signal transduction, GSK3, JNK, PP1

## Abstract

Tau protein is subject to phosphorylation by multiple kinases at more than 80 different sites. Some of these sites are associated with tau pathology and neurodegeneration, but other sites are modified in normal tau as well as in pathological tau. Although phosphorylation of tau at residues in the microtubule-binding repeats is thought to reduce tau association with microtubules, the functional consequences of other sites are poorly understood. The AT8 antibody recognizes a complex phosphoepitope site on tau that is detectable in a healthy brain but significantly increased in Alzheimer’s disease (AD) and other tauopathies. Previous studies showed that phosphorylation of tau at the AT8 site leads to exposure of an N-terminal sequence that promotes activation of a protein phosphatase 1 (PP1)/glycogen synthase 3 (GSK3) signaling pathway, which inhibits kinesin-1-based anterograde fast axonal transport (FAT). This finding suggests that phosphorylation may control tau conformation and function. However, the AT8 includes three distinct phosphorylated amino acids that may be differentially phosphorylated in normal and disease conditions. To evaluate the effects of specific phosphorylation sites in the AT8 epitope, recombinant, pseudophosphorylated tau proteins were perfused into the isolated squid axoplasm preparation to determine their effects on axonal signaling pathways and FAT. Results from these studies suggest a mechanism where specific phosphorylation events differentially impact tau conformation, promoting activation of independent signaling pathways that differentially affect FAT. Implications of findings here to our understanding of tau function in health and disease conditions are discussed.

## Introduction

Tau is a neuronal microtubule-associated protein enriched in axons, which becomes abnormally phosphorylated and aggregated in a group of neurodegenerative diseases including Alzheimer’s disease (AD), collectively known as tauopathies (Wang and Mandelkow, 2016). Due to early work showing tau promotes microtubule assembly *in vitro*, the major functional role of tau has been thought to be microtubule stabilization (Weingarten et al., 1975). However, tau has a highly dynamic secondary structure and post-translational modifications, including phosphorylation, might stabilize specific conformations. Furthermore, a wide variety of observations support additional roles for this protein, including regulation of intracellular organelle trafficking through modulation of phosphotransferases (Kanaan et al., 2013; Kneynsberg et al., 2017). Such roles appear consistent with findings of tau interactions with various protein kinases and phosphatases (Lee et al., 1998; Liao et al., 1998; Sun et al., 2002; Sontag et al., 2012).

In its soluble monomeric form, WT tau displays a highly dynamic secondary structure (Melková et al., 2019) but the monomers mainly adopt a “paperclip” conformation, where the N- and C-terminals fold in towards the central microtubule-binding domains (Jeganathan et al., 2006). Tau is a major phosphoprotein in the brain, with over 80 potential phosphorylation sites. Of those, approximately 50 may be phosphorylated in normal development and/or disease conditions (Šimić et al., 2016). Phosphorylation has been shown to impact tau conformation with pseudophosphorylation of the AT8 (pS199, pS202, pT205) or PHF1 (pS396, pS404) antibody epitopes promoting “open” conformations where both the N- and C-termini are displaced away from the central microtubule-binding domains (Jeganathan et al., 2008; Bibow et al., 2011; Kanaan et al., 2011; Combs and Kanaan, 2017). However, several questions remain about the effects of phosphorylation of tau at specific sites. Phosphorylated sites within the AT8 antibody epitope (pS199, pS202, pT205) are of particular interest, as these sites are detected in normal brain tissues and widespread AT8 immunoreactivity is frequently used for the diagnosis of tauopathies (Goedert et al., 1993; Watanabe et al., 1993; Maurage et al., 2003). Moreover, the question of how these conformational changes may affect tau function other than microtubule-binding has not been addressed.

Our prior work has linked conformation-dependent effects of tau on fast axonal transport (FAT), a cellular process involving bidirectional translocation of organelles along axons (Kneynsberg et al., 2017). Anterograde FAT delivers organelle cargoes carried by members of the kinesin superfamily of motor proteins, away from the cell body towards the synapse, whereas cytoplasmic dynein carries other cargoes in the retrograde direction (Morfini et al., 2016; Brady and Morfini, 2017). Studies in the isolated squid axoplasm model showed that monomeric tau pseudophosphorylated at all three S/T residues in the AT8 epitope (hTau40-AT8) selectively inhibited kinesin-1-based anterograde FAT (Kanaan et al., 2011). This inhibitory effect was associated with aberrant exposure of an 18 amino acid biologically active domain at the amino terminus of tau termed the phosphatase activating domain (PAD). Increased PAD exposure leads to activation of a protein phosphatase 1 (PP1)-glycogen synthase 3β (GSK3β) signaling pathway, which in turn promotes detachment of kinesin-1 from its transported organelle cargoes (Kanaan et al., 2011). Intriguingly, there is considerable overlap between tau phosphospecies found in the context of tauopathies and those seen in normal developing or adult brains, including the AT8 epitope (Matsuo et al., 1994; Kimura et al., 2016b) suggesting these tau phosphospecies may have a normal function in the regulation of FAT.

Collectively, the observations above prompted us to evaluate whether phosphorylation of monomeric tau at selected residues within the AT8 epitope (S199, S202, T205 in hTau40) suffice to confer upon tau a modulatory effect(s) on FAT. Surprisingly, we found that pseudophosphorylation of selected residues sufficed for monomeric tau to inhibit either anterograde FAT or both anterograde and retrograde FAT. Additionally, we present evidence linking these effects to PAD exposure and activation of selected kinase pathways. Collectively, findings here are consistent with a mechanism linking residue-specific phosphorylation of tau, stabilization of unique conformations, and activation of selected phosphotransferase signaling pathways. Implications of these findings to disease and developmental-related roles of tau are discussed.

## Materials and Methods

### Reagents and Antibodies

The following antibodies were used for Western blots: KHC (H2 clone; Pfister et al., 1989); Total GSK3 (Cell Signaling Technology #D75D3); pJNK (Cell Signaling Technology #9251). Anti-dp-ser9/21-GSK3α/β (clone 15C2; Grabinski and Kanaan, 2016) and anti-tau (clone TNT1) were provided by Dr. Nicholas Kanaan (Michigan State University).

### Recombinant Tau Proteins

**Figure 1:**
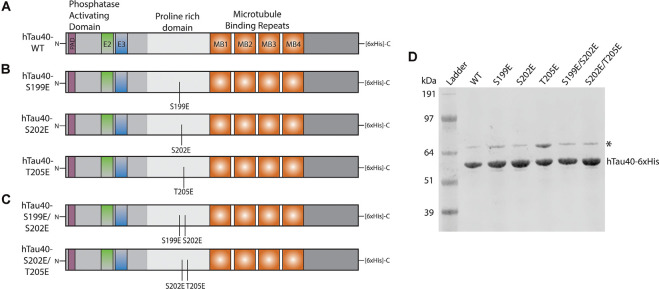
Schematic of recombinant tau proteins used. **(A)** hTau40-WT corresponds to the longest isoform of human CNS tau containing two N-terminal inserts (exons 2 and 3) and four microtubule-binding repeats. **(B)** The hTau40-S199E, hTau40-S202E, and hTau40-T205E recombinant proteins were each pseudophosphorylated by a single point mutation (S$→$E or T$→$E) of the AT8 epitope. **(C)** The hTau40-S199E/S202E and hTau40-S202E/T205E recombinant proteins were pseudophosphorylated at two combinations of the individual mutation sites. **(D)** Each recombinant tau protein was run on SDS–PAGE (1 μg/lane) and the gel was stained with Coomassie to show each protein. DnaK (~70 kDa) copurifies with tau protein (Combs et al., 2017).

Recombinant tau proteins in Figure 1 were prepared as previously described (Tiernan et al., 2016; Combs et al., 2017). Single or multiple pseudophosphorylation point mutations were introduced into a cDNA construct encoding full-length hTau40 using Quikchange II XL Site-Directed Mutagenesis Kit (Agilent) and confirmed by Sanger sequencing. All constructs included a C-terminal 6× Histidine tag to aid protein purification. For protein expression, cDNA constructs encoding WT or pseudophosphorylated hTau40 were transformed in Rosetta2 competent cells (Novagen) and starter cultures (100 ml) were incubated overnight at 37°C with shaking before being used to inoculate TB with 0.2% glucose (1 L). Cultures were incubated for a further 3 h to an OD_600_ ~0.8 at which time IPTG (Isopropyl β-D-1-thiogalactopyranoside) was added to a final concentration of 0.4 mM to induce protein expression. After 3 h, bacteria pellets were collected by centrifugation at 8,000 *g* and resuspended in lysis buffer (500 mM NaCl, 10 mM Tris, 5 μM Imidazole) with protease inhibitor cocktail (Sigma–Aldrich). Cells were sonicated on ice and tau was purified from cleared lysates with Talon Metal Affinity Resin as per the manufacturer’s instructions (Takara Bio). Eluted proteins were concentrated and buffer exchanged into 50 mM HEPES, 100 mM KCl using Amicon 30 kDa columns (Millipore).

### Squid Axoplasm Vesicle Motility Assays

Axoplasms were extruded from giant axons of the squid *Loligo pealii* (Marine Biological Laboratory) as described (Song et al., 2016). Tau recombinant proteins were diluted into buffer X/2 (175 mM potassium aspartate, 65 mM taurine, 35 mM betaine, 25 mM glycine, 10 mM HEPES, 6.5 mM MgCl_2_, 5 mM EGTA, 1.5 mM CaCl_2_, 0.5 mM glucose, pH 7.2) supplemented with 5 mM ATP and perfused into isolated squid axoplasm at final concentration of 2 μM. For TNT1 blocking experiments (Figure 3), tau recombinant proteins were incubated with 3 mg/ml TNT1 for 1 h on ice before the addition of buffer X/2 and perfusion. Vesicle motility was analyzed on a Zeiss Axiomat with a 100×, 1.3 N.A. objective, and differential interference contrast optics. Images were acquired using a Model C2400 CCD through a Hamamatsu Argus 20 and further process using a Hamamatsu Photonics Microscopy C2117 video manipulator for image adjustment and generation of calibrated cursors and scale bars. Anterograde and retrograde FAT rates were measured by matching calibrated cursor movements to the speed of vesicles moving in the axoplasm over 50 min. FAT rates were plotted as a function of time.

**Figure 2:**
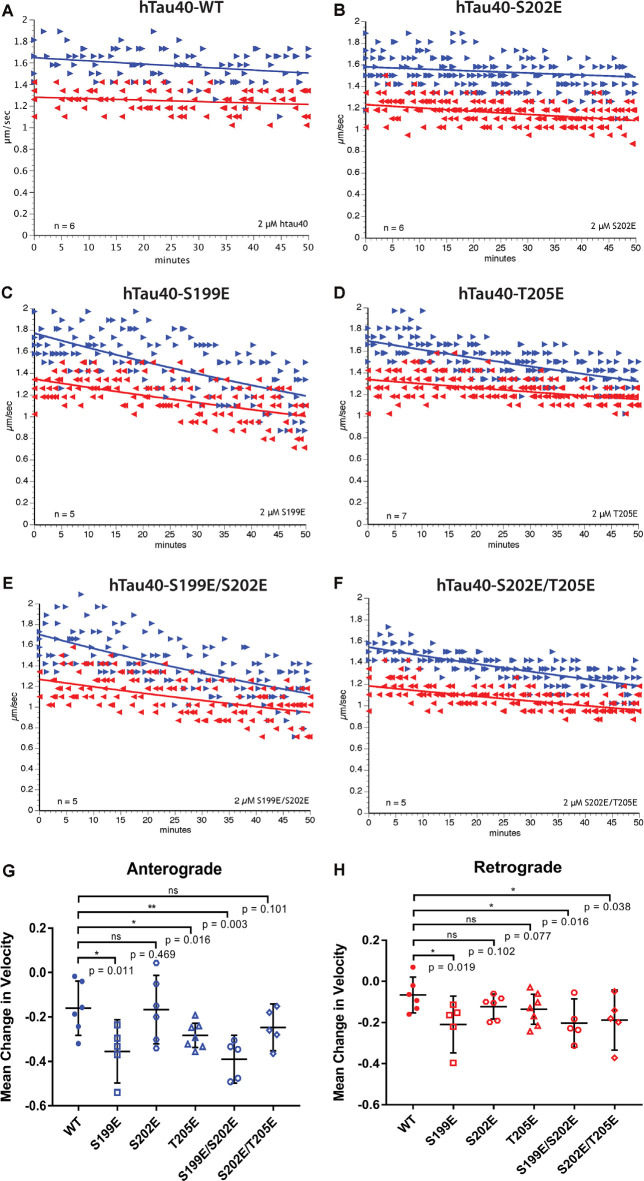
Differential phosphorylation of tau within the AT8 epitope can inhibit either anterograde or both directions of fast axonal transport (FAT). **(A–F)** The vesicle motility assay in isolated squid axoplasm is an *ex vivo* model system that allows for simultaneous, quantitative analysis of anterograde and retrograde FAT (Song et al., 2016). Graphs are plotted as velocity (μm/s) against time with right arrows ($▸$) indicating anterograde data points and left arrows ($◂$) indicating retrograde data points. **(A)** Perfusion of WT tau monomers did not affect the rate of axonal transport in either the anterograde or retrograde direction. **(B)** hTau40-S202E monomers were not sufficient to inhibit FAT in either the anterograde or retrograde directions. **(C)** In contrast, Tau monomers pseudophosphorylated at serine 199 (hTau40-S199E) inhibited FAT in both directions, whereas **(D)** pseudophosphorylation of tau monomers at threonine 205 (hTau40-T205E) selectively inhibited anterograde FAT. **(E)** Pseudophosphorylation of tau monomers at serine 199 and serine 202 (hTau40-S199E/S202E) inhibited FAT in both directions whereas **(F)** tau monomers pseudophosphorylated at serine 202 and threonine 205 (hTau40-S202E/T205E) inhibited retrograde FAT with a trend towards inhibition of anterograde FAT. **(G)** Quantitative analysis of FAT demonstrated that S199E (*n* = 5; *p* = 0.011), T205E (*n* = 7; *p* = 0.016), and S199E/S202E (*n* = 5; *p* = 0.003) significantly inhibited anterograde FAT compared to hTau40-WT (*n* = 6). By contrast, S202E (*n* = 6, *p* = 0.469) and S202E/T205E (*n* = 5; *p* = 0.101) were not significantly different from hTau40-WT, although anterograde FAT showed a tendency for reduction with S202E/T205E that may become significant after longer incubation. **(H)** Compared to hTau40-WT, retrograde FAT was significantly inhibited by S199E (*n* = 5; *p* = 0.019), S199E/S202E (*n* = 5; *p* = 0.016), and S202E/T205E (*n* = 5; *p* = 0.038) but was unaffected by S202E (*p* = 6; *p* = 0.102) and T205E (*n* = 7; *p* = 0.077). All scatter plots are presented as mean ± 95% confidence interval.

**Figure 3:**
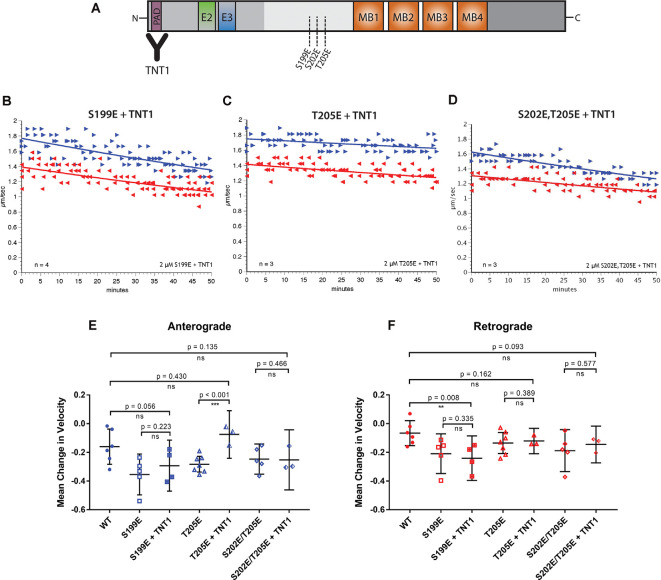
Specific inhibition of anterograde FAT can be blocked by incubation with a PAD antibody. **(A)** Pseudophosphorylated tau monomers were incubated with TNT1 antibody which specifically binds the PAD (Kanaan et al., 2011) for 1 h before perfusion into the isolated squid axoplasm. **(B)** Even after incubation of hTau40-S199E with TNT1 FAT was still inhibited in both directions. **(C)** Incubation of TNT1 with hTau40-T205E prevented the specific inhibition of anterograde FAT. **(D)** Co-perfusion of TNT1 with hTau40-S202E/T205E did not prevent inhibition of FAT. **(E)** Quantitative analysis of anterograde FAT revealed no significant difference between hTau40-S199E perfused with (*n* = 4) or without (*n* = 5) incubation with TNT1 (*p* = 0.223). By contrast, hTau40-T205E + TNT1 (*n* = 3) was comparable to hTau40-WT (*n* = 5; *p* = 0.430) and was significantly different to hTau40-T205E without TNT1 (*n* = 7; *p* < 0.001). There was no significant difference between hTau40-S202E/T205E alone (*n* = 5) or hTau40-S202E/T205E + TNT1 (*n* = 4; *p* = 0.466). **(F)** hTau40-S199E + TNT1 (*n* = 3) still significantly inhibited retrograde FAT compared to hTau40-WT (*n* = 5; *p* = 0.008). hTau40-T205E (*n* = 7) did not inhibit retrograde FAT and there was no change with TNT1 (*n* = 3; *p* = 0.389). hTau40-S202E/T205E + TNT1 (*n* = 3) was not significantly different from hTau40-S202E/T205E alone (*n* = 4; *p* = 0.577). All scatter plots are presented as mean ± 95% confidence interval.

### Immunoblotting-Based Analysis of Kinase Activity in Squid

Lysates were prepared from “sister” axoplasms as described (Kang et al., 2016). Briefly, two axons from an individual squid were dissected and extruded onto glass slides. One axoplasm was perfused with a Control perfusion mix (Buffer X/2) and the other with an Experimental perfusion mix (5 μM Tau recombinant protein). After 50 min incubation, axoplasms were collected in 1% SDS and 6× sample buffer in preparation for immunoblotting.

Squid axoplasm lysates were run on 4–12% Bis/Tris gels (Invitrogen) in MOPS buffer and proteins were transferred to PVDF membrane (Bio-Rad) using Towbin buffer with 10% methanol. Membranes were dried for at least 1 h before being reactivated in methanol and blocked in 1% milk in TBS supplemented with 2 mM Sodium Orthovanadate and 10 mM Sodium Fluoride to block phosphatase activity present in milk. Primary antibodies were incubated overnight diluted in 1% BSA in TBS supplemented as before. Goat anti-mouse or goat anti-rabbit secondary antibodies (LiCor) were incubated for 1 h at RT and immunoblots visualized using a LiCor Odyssey Fc imaging system. LiCor ImageStudio Lite was used for quantitation of signal intensity.

### Statistics

All experiments were repeated at least three times (specific *n* values shown in Figures). For vesicle motility assays, the difference between the mean of velocity measurements obtained between 0–10 and 30–50 min after perfusion was calculated for both anterograde and retrograde FAT in each experiment. For biochemistry experiments, signal intensities of “sister” axoplasms were quantified as described in Section “Immunoblotting-Based Analysis of Kinase Activity in Squid” (Kang et al., 2016). Experiments were analyzed by unpaired (vesicle motility assay) or paired (biochemistry) Student’s *t*-test. Statistical tests were carried out in Graphpad Prism 7 software and significance was at *p* < 0.05 for all experiments.

## Results

### Pseudophosphorylated Tau Proteins Can Inhibit Axonal Transport

Tau is a well-documented phosphoprotein that undergoes phosphorylation at multiple potential sites *in vivo* (Šimić et al., 2016). Among several well-characterized phosphorylation sites, there is considerable overlap of tau residues phosphorylated during development and in tauopathies (Brion et al., 1993; Goedert et al., 1993; Yu et al., 2009). To date, the functional significance of specific phosphorylation events in tau has mainly been limited to measuring their impact on aggregation or microtubule-binding, without consideration of other potential functions. One exception is the AT8 epitope, comprising a set of phosphorylation sites prominent both in AD and during development (Matsuo et al., 1994; Kimura et al., 2016b). Previously, we evaluated the effect of triply pseudophosphorylated hTau-AT8 on FAT using vesicle motility assays in the isolated squid axoplasm preparation (Kanaan et al., 2011). This assay is a well-characterized *ex vivo* model system that allows for quantitative analysis of anterograde and retrograde FAT (Song et al., 2016). When perfused in squid axoplasm hTau-AT8 monomers specifically inhibited anterograde, kinesin-1-dependent FAT through a mechanism involving exposure of PAD and activation of a PP1-GSK3β pathway, which in turn promoted detachment of the anterograde FAT motor kinesin-1 from its transported cargoes (Morfini et al., 2002, 2004; LaPointe et al., 2009; Kanaan et al., 2011).

The findings above established a mechanism linking a specific pathological tau species (tau triply phosphorylated at the AT8 epitope) to PAD exposure and activation of a downstream PP1-GSK3β signaling pathway. However, ample data exists showing that some tau phosphospecies are detectable in the normal and pathological brain including phosphorylation of some, but not all three S/T residues in the AT8 antibody epitope. For example, pS202 and pT205 tau are detected in cerebrospinal fluid of AD patients, with pT205 showing the highest correlation with disease progression (Barthélemy et al., 2020). Additionally, tau phosphorylated at S199 and S202 residues are enriched in the somatodendritic compartment, whereas tau phosphorylated at T205 mainly localizes to axons (Binder et al., 1985; Hernández et al., 2003). Based on these precedents we examine more closely whether defined phosphorylation sites in the AT8 epitope, individual or in two combinations, suffice to confer upon WT tau a modulatory effect(s) on FAT.

Pseudophosphorylated forms of recombinant tau proteins were generated featuring triple mutation of serine and threonine residues within the AT8 epitope (S199E, S202E, T205E) to glutamic acid, which mimics the negative charge effect associated with phosphorylation (Figure 1). Following expression in bacteria, purified before being tested using vesicle motility assays in the squid axoplasm preparation (Figure 2). As shown in Figure 2A, perfusion of htau40-WT monomers did not affect either anterograde or retrograde FAT, a result consistent with our prior work (Morfini et al., 2007; LaPointe et al., 2009; Kanaan et al., 2011). Similarly, hTau40-S202E did not affect FAT rates in either the anterograde (Figures 2B,G) or retrograde (Figures 2B,H) directions. By contrast, htau40-T205E selectively inhibited anterograde (Figures 2D,G), but not retrograde FAT (Figures 2D,H), much as reported for AT8 tau (S199E/S202E/T205E; Kanaan et al., 2011). Surprisingly, hTau40-S199E was found to inhibit both anterograde (Figures 2C,G) and retrograde FAT (Figures 2C,H) an effect previously observed with aggregated hTau40-S422E (Tiernan et al., 2016). Taken together, these results revealed that single pseudophosphorylation of tau at either S199 or T205 residues sufficed to confer upon monomeric tau differential effects on FAT and tau conformation, whereas phosphorylation at S202 did not.

Next, we set out to determine whether the effects elicited by hTau40-T205E and hTau40-S199E on FAT were impacted by phosphorylation of the adjacent S202 residue. As shown in Figures 2E,G,H, hTau40-S199E/S202E significantly inhibited FAT in both directions, like hTau40-S199E. By contrast, hTau40-S202E/T205E significantly inhibited retrograde FAT (Figures 2F,H). Although the effects of hTau40-S202E/T205E on anterograde FAT did not reach significance, there was a clear trend towards reduced rates that may have reached significance with longer incubation (Figure 2G). Thus, while pseudophosphorylation of the S202 residue did not change the effects of hTau40-S199E on either FAT direction, it modulated the effects of hTau40-T205E on FAT and tau conformation.

### Differential Effects of Pseudophosphorylated Tau Proteins on Fast Axonal Transport Involve PAD-Dependent and PAD-Independent Pathways

Exposure of PAD in tau, an event associated with various pathological post-translational modifications, has been shown to trigger a PP1-GSK3β pathway that inhibits anterograde FAT (Kanaan et al., 2011). To explore whether inhibition of FAT elicited by pseudophosphorylated tau proteins involves PAD exposure, we performed co-perfusion experiments (Figure 3). Specifically, we evaluated the ability of the TNT1 antibody, which binds to PAD (Figure 3A; Kanaan et al., 2011) to prevent inhibitory effects of hTau40-S199E, hTau40-T205E, and hTau40-S202E/T205E on FAT. Recombinant tau proteins were preincubated with TNT1 antibody for 1 h before perfusion into the isolated squid axoplasm for the vesicle motility assay (Figure 3A). Perfusion of TNT1 antibody alone did not affect either direction on FAT (data not shown).

Co-perfusion of TNT1 with hTau40-T205E prevented inhibition of anterograde FAT by this protein (Figures 3C,E). By contrast, co-perfusion with TNT1 failed to prevent the inhibitory effect of hTau40-S199E in either anterograde (Figures 3B,E) or retrograde (Figures 3B,F) directions. These results indicated that exposure of the PAD is necessary for hTau40-T205E to inhibit anterograde FAT and that hTau40-S199E inhibits both directions of FAT through a PAD-independent mechanism. Co-perfusion of TNT1 antibody did not change the effects of hTau40-S202E/T205E on FAT, which mildly inhibited anterograde FAT and significantly inhibited retrograde FAT, indicating that pseudophosphorylation at both of these sites in the same protein does not promote PAD exposure, and suggested that modifying both S202 and T205 induces a different conformation of tau than modification of T205 only.

### Differential Effects of htau40-T205E and htau40-S199E on FAT Involve Activation of Distinct Kinase Signaling Pathways

**Figure 4:**
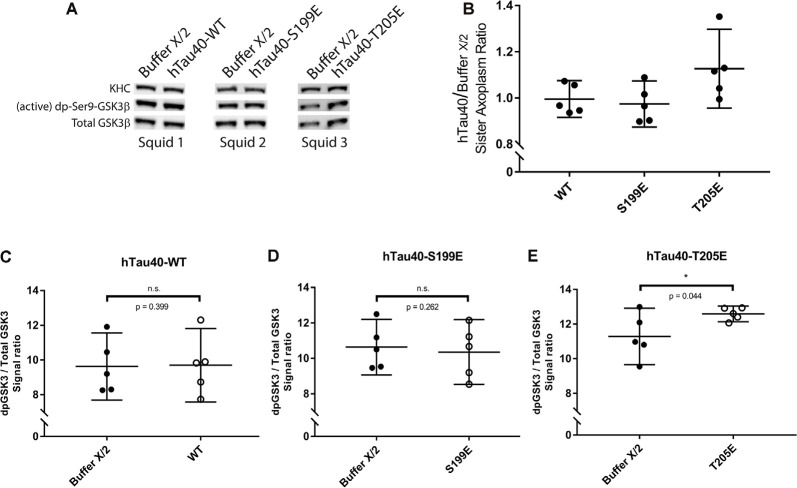
hTau40-T205E activates GSK3β. **(A)** Representative images showing immunoblots of lysates from three “sister” axoplasm pairs, each obtained from a single independent squid (squids 1–3; Kang et al., 2016). Each “sister” axoplasm pair was perfused with either Buffer X/2 (control) vs. hTau40-WT monomers, Buffer X/2 vs. hTau40-S199E monomers, or Buffer X/2 vs. hTau40-T205E monomers. After a 50-min incubation, axoplasm lysates were prepared, separated by SDS–PAGE and immunoblots revealed using antibodies recognizing (active) dephosphorylated-ser9-GSK3β and total GSK3β. **(B)** Quantitation of dpGSK3β immunoblot signal (normalized to total GSK3β) for “sister” axoplasm pairs (hTau40/Buffer X/2; *n* = 5 axoplasm pairs for each hTau40 pseudophosphorylated protein). **(C–E)** Quantification of active dp-GSK3β immunoblot signal normalized to total GSK3β revealed that perfusion of hTau40-T205E monomers led to more active dp-GSK3β compared to Buffer-X perfused sister axoplasms (*n* = 5; *p* = 0.044; **E**). Perfusion of hTau40-WT (*n* = 5; *p* = 0.399; **C**) and hTau40-S199E (*n* = 5; *p* = 0.261; **D**) did not result in more active dp-GSK3β.All data are presented as mean ± 95% confidence interval.

Exposure of PAD leads to activation of GSK3β, which in turn inhibits anterograde FAT by phosphorylating light chain subunits of kinesin-1 (Morfini et al., 2002; Kanaan et al., 2011). Results from co-perfusion experiments suggested that hTau40-T205E may also act through this pathway (Figures 3C,E). To evaluate this possibility, we performed immunoblotting experiments in “sister” axoplasms perfused with either buffer X/2 alone (control) or with hTau40-T205E (diluted in Buffer X/2). Immunoblots were developed using a well-characterized antibody that selectively recognizes active GSK3β species dephosphorylated at the regulatory serine 9 residue (anti-dpS9-GSK3β; Figure 4). Dephosphorylation of this critical regulatory residue in GSK3β by any of several protein phosphatases, including PP1, represents a major activation mechanism for this kinase (Grabinski and Kanaan, 2016). A phosphorylation-independent antibody against GSK3β provided an internal protein loading control (anti-Total GSK3β). Due to variation in baseline kinase activation among different squid, all comparisons were pairwise between sister axoplasms (Figure 4A; Kang et al., 2016), visualized in Figure 4B (hTau40/Buffer X/2). Perfusion of hTau40-WT (Figures 4B,C) or hTau40-S199E (Figures 4B,D) did not affect GSK3β activity. In contrast, perfusion of hTau40-T205E tau caused a statistically significant increase in anti-dpGSK3β/anti-Total GSK3β immunoreactivity ratios, indicative of GSK3β activation (Figure 4E).

**Figure 5:**
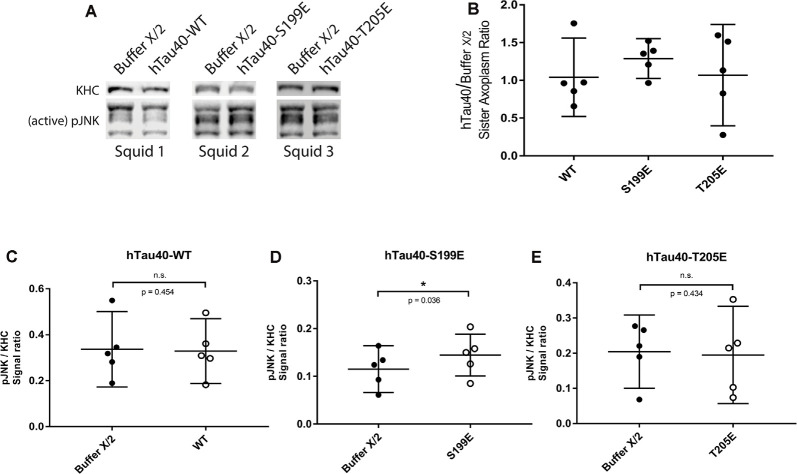
hTau40-S199E activates pJNK. **(A)** Representative images showing immunoblots of lysates from three “sister” axoplasm pairs, each pair obtained from a single independent squid (squids 1–3; Kang et al., 2016). Each “sister” axoplasm was perfused with either Buffer X (control) vs. hTau40-WT monomers, Buffer X vs. hTau40-S199E monomers, or Buffer X vs. hTau40-T205E monomers. After a 50-min incubation, axoplasm lysates were prepared, separated by SDS–PAGE and immunoblots revealed using antibodies that recognize phosphorylated-active forms of JNK kinases (pJNK), and with a monoclonal antibody that recognizes heavy chain subunits of the motor kinesin-1 (KHC, internal control for total axoplasmic protein loading). **(B)** Quantitation of pJNK immunoblot signal (normalized to KHC) for “sister” axoplasm pairs (hTau40/Buffer X/2; *n* = 5 axoplasm pairs for each hTau40 pseudophosphorylated protein). **(C–E)** Quantification of pJNK immunoblots. Perfusion of hTau40-S199E significantly increased pJNK activation compared to Buffer X (*n* = 5; *p* = 0.037; **D**) whereas hTau40-WT (*n* = 5; *p* = 0.453; **C**) or hTau40-T205E (*n* = 5; *p* = 0.434; **E**) had no overall effect. pJNK signal intensity was normalized to kinesin heavy chain (KHC). All data are presented as mean ± 95% confidence interval.

When perfused in axoplasm, hTau40-S199E inhibited both directions of FAT and these effects were not rescued by TNT1 co-perfusion (Figures 3B,E,F). Prior work revealed several kinases that inhibit both directions of FAT, including selected JNK (JNK3) and p38 kinase (p38β) isoforms (Brady and Morfini, 2017). Interestingly, JNKs are well known to be activated in AD (Pei et al., 2001; Zhu et al., 2001; Yarza et al., 2015) and were previously implicated as a downstream effector of trafficking defects in tau knockout Drosophila (Voelzmann et al., 2016). Based on these precedents, lysates prepared from sister axoplasms perfused with hTau40-WT and hTau40-S199E were analyzed for activation of JNK kinases by immunoblotting with an antibody that recognizes phosphorylated, catalytically active JNKs (pJNK). An antibody that recognizes heavy chain subunits (KHC) of squid kinesin-1 (Brady et al., 1990) was used for normalization purposes (Figure 5A). As shown in Figures 5B,D, hTau40-S199E perfusion significantly increased pJNK/KHC immunoreactivity ratios, whereas hTau40-WT (Figures 5B,C) or hTau40-T205E (Figures 5B,E) did not. Taken together, these experiments indicate activation of different downstream kinase pathways by hTau40-T205E and hTau40-S199E in a manner dependent and independent of PAD, respectively.

## Discussion

Tau was originally identified through its interactions with microtubules (Weingarten et al., 1975). Subsequent studies on tau function naturally focused on microtubule binding. Studies on the role of tau in AD and other tauopathies established a high level of phosphorylation for tau as a major histopathological hallmark (Wang and Mandelkow, 2016). Consistent with the focus on microtubule binding, increased phosphorylation of tau in pathological states correlated with reduced binding to microtubules. However, tau has more than 80 sites that can be phosphorylated and most of these sites are not associated with the microtubule-binding repeats (Šimić et al., 2016). This raised the question of how tau phosphorylation at sites that are heavily phosphorylated in pathological tau affects tau functions or structure.

Previously, we reported that triple pseudophosphorylation at the AT8 epitope (S199, S202, T205) led to a conformational change in tau that exposed a biologically active motif in the N-terminal of tau that is normally sequestered in dephosphorylated tau (Kanaan et al., 2011). This sequence (the Phosphatase Activating Domain or PAD) activates a PP1/GSK3β signaling pathway resulting in specific inhibition of anterograde FAT (Morfini et al., 2004; LaPointe et al., 2009; Kanaan et al., 2011). PAD undergoes aberrant exposure during early disease stages in tauopathies, in a manner concomitant with progressive tau aggregation and phosphorylation at the AT8 epitope (Kanaan et al., 2011, 2016; Combs et al., 2016). These studies provided a potential mechanism linking pathological forms of tau to deficits in FAT and axonal pathology in the context of AD and other tauopathies (Kneynsberg et al., 2017). However, tau also undergoes extensive phosphorylation during development, including at selected sites within the AT8 epitope (Goedert et al., 1993; Watanabe et al., 1993). *In vivo*, the three residues within the AT8 epitope (S199, S202, T205) can be differentially phosphorylated either singly or in different combinations (Binder et al., 1985; Maurage et al., 2003; Barthélemy et al., 2019). Based on these precedents, we sought to evaluate the effects of phosphorylation of tau at single and doubly phosphorylated residues in the AT8 epitope had any effect(s) on FAT.

**Figure 6:**
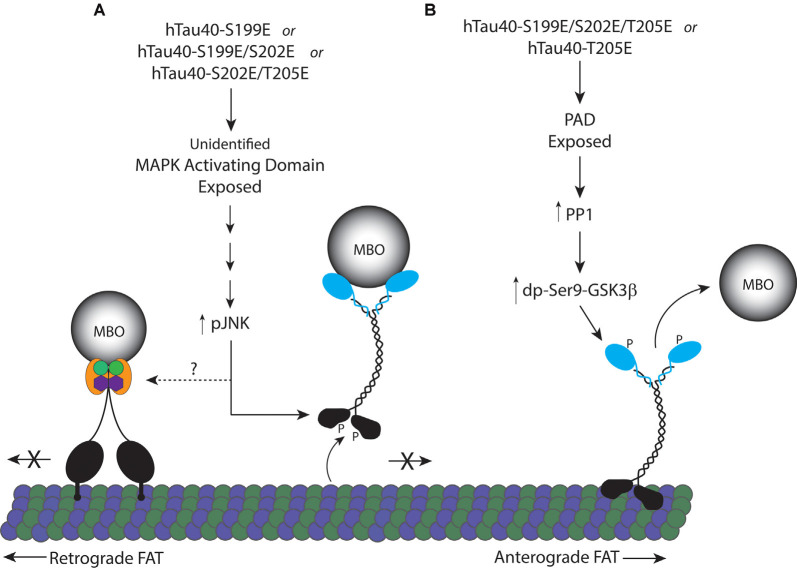
Proposed model for inhibition of FAT by pseudophosphorylated tau proteins. **(A)** Tau monomers individually pseudophosphorylated at S199 (hTau40-S199E), or doubly pseudophosphorylated at S199 and S202 (hTau40-S199E/S202E), or S202 and T205 (hTau40-S202E/T205E) fold into a conformation that exposes an unidentified MAPK activating domain leading to activation of pJNKs which can directly phosphorylate kinesin-1 heavy chain subunits inhibiting their association with microtubules (Morfini et al., 2009b). JNK3 also inhibits retrograde FAT through an unknown mechanism. **(B)** Pseudophosphorylation of tau monomers at T205 (hTau40-T205E) or S199, S202 and T205 (hTau40-S199E/S202E/T205E; Kanaan et al., 2011) fold into a conformation that exposes the N-terminal PAD resulting in activation of a PP1/GSK3β signaling cascade that phosphorylates kinesin light chain ending in release of the cargo from kinesin.

Despite the proximity of the three phosphorylation sites analyzed here, we found that single phosphorylation at S199 or T205, but not at S202, produces monomeric tau that differentially affects FAT by activation of two distinct downstream signaling pathways. Specifically, hTau40-T205E inhibited anterograde FAT through a mechanism involving exposure of the N-terminal PAD and PP1/GSK3β activation, as seen with hTau40-AT8 in prior studies (Kanaan et al., 2011). By contrast, perfusion of hTau40-S199E inhibited FAT in both directions, an effect previously seen with aggregated hTau40-S422E (Tiernan et al., 2016). In a model that encompasses the data here (Figure 6), phosphorylation of monomeric tau at specific residues induces distinct conformations that increase the exposure of the PAD or another yet to be determined biologically active domain(s). In turn, these domains differentially trigger activation of a PP1-GSK3β pathway or a MAPK pathway leading to JNK kinases. The differences in downstream effects of individually pseudophosphorylated monomeric tau indicate a high degree of complexity in the manner by which phosphorylation promotes conformational change(s) and alters tau function.

Tau is generally classified as an intrinsically disordered protein. However, multiple studies have indicated that monomeric tau normally folds into a “paperclip” conformation with the N- and C-terminals near the central microtubule-binding domains (Jeganathan et al., 2008; Bibow et al., 2011; Di Primio et al., 2017). *In vitro*, pseudophosphorylation of the AT8 epitope exposes the N-terminal whereas pseudophosphorylation at the C-terminal PHF1 epitope promotes exposure of the C-terminal (Jeganathan et al., 2008). Our results suggest that the relationship between tau’s phosphorylation state and its conformation is more complex than a simple opening up of the N- or C- terminals, as three closely linked phosphorylation sites confer upon tau distinct effects on FAT and kinase-based signaling. Pseudophosphorylation at T205E effects similar to the effects of triple phosphorylation (Kanaan et al., 2011), exposing the N-terminal PAD and activating a PP1/GSK3 signaling pathway that is blocked by TNT1. In contrast, S199E inhibited both anterograde and retrograde FAT which could not be rescued by TNT1, indicating that the PAD exposure was not responsible for these effects. Instead, the results suggest that hTau40-S199E folds into a conformation that led to the activation of JNK kinases. Interestingly, some JNK isoforms mimic the effects of hTau40-S199E on FAT, also inhibiting both anterograde and retrograde FAT (Morfini et al., 2009a; Brady and Morfini, 2017). The mechanism by which tau can activate JNK remains to be elucidated, but tau contains numerous PXXP motifs, which can bind to SH3 motifs that are a common feature of selected MAP3Ks upstream of JNK (Gallo and Johnson, 2002). Consistent with this idea, tau is known to interact with several SH3 domain proteins in a phosphorylation-dependent manner (Reynolds et al., 2008; Usardi et al., 2011; Sottejeau et al., 2015).

Investigation of dually pseudophosphorylated hTau40 proteins indicated additional levels of complexity. Both hTau40-S199E/S202E and hTau40-S199E similarly inhibited both directions of FAT, implying that S202 pseudophosphorylation does not alter the specific hTau40 conformation elicited by S199 pseudophosphorylation significantly. In contrast, pseudophosphorylation of S202 significantly changed the effects of hTau40-T205E. Whereas hTau40-T205E affected only anterograde FAT and the effect was blocked by TNT1, hTau40-S202E/T205E inhibited retrograde FAT and exhibited a slow decline in anterograde FAT that did not reach significance in 50 min. Co-perfusion with TNT1 did not change the effects on either anterograde or retrograde FAT by hTau40-S202E/T205E. Tau is known to have a dynamic secondary structure, which can be stabilized by phosphorylation of selected residues (Jeganathan et al., 2008; Bibow et al., 2011). Our results suggest hTau40-S202E/T205E may have a more dynamic structure than other pseudophosphorylated hTau40 proteins tested, and the possibility remains that this may reflect still another conformation and signaling pathway.

Although most studies on tau function have focused on microtubule dynamics, there is evidence that tau can also affect neuronal signaling in other ways. For example, tau has also been suggested to potentiate NGF-ERK signal transduction in differentiating PC12 cells specifically through a mechanism involving phosphorylation at T231. Tau depleted cells were unable to initiate neurite extension and the effect could be rescued by exogenous expression of WT but not Tau-T231A (Leugers and Lee, 2010). Interestingly, expression of Tau-S199D/S202D actually decreased activation of ERK in response to NGF compared to WT tau demonstrating that signal transduction through tau phosphorylation depends on the site modified and the function examined (Leugers et al., 2013). Other studies have suggested that phosphorylation at S396/404 is essential for hippocampal LTD although it has not been determined whether this leads to downstream signal transduction or involves some other mechanism (Kimura et al., 2014b). Combined with our results, these studies strongly suggest that tau may function as a scaffolding protein. Taken together, the various reports documenting an association of tau with selected protein kinases and phosphatases, in combination with data here, provide strong evidence that tau functions as a “signaling hub,” playing a role in regulating signaling pathways by scaffolding and modulation of phosphotransferase activity in a phosphorylation-dependent manner.

While tau phosphorylation has commonly been studied in the context of disease, various lines of evidence indicate that it also has additional physiological functions, especially important during development. The relative levels of tau phosphorylation and the specific sits modified change during the development and maturation of the brain. When tau from the fetal brain is analyzed for the amount of phosphorylation, it has approximately six phosphates per molecule of tau whereas in tau from the adult human brain the ratio is 2–3 mol of phosphate per mol of protein (Kenessey and Yen, 1993; Köpke et al., 1993). However, these are averages for phosphorylation levels in total tau and the phosphates are unlikely to be split evenly between all tau protein, so a given pool of tau may bear phosphorylation at a few sites while others may have levels much higher than the average. Studies using PhosTag gels of adult mouse tau have found that a large fraction of tau had no detectable phosphorylation (Kimura et al., 2016a). Moreover, tau has long been known to be differentially phosphorylated in different neuronal domains. For example, the Tau1 antibody recognizes tau that is unphosphorylated between S195 to S202, and Tau1 immunoreactivity is primarily seen in axons (Binder et al., 1985; Papasozomenos and Binder, 1987), while tau antibodies to epitopes that are not subject to phosphorylation detect tau in both axonal and somatodendritic domains. Correspondingly, tau phosphorylated at pS199 is enriched in the somatodendritic region in both young and old healthy brains (Maurage et al., 2003). Although our study found that perfusion of hTau40-S199E led to inhibition of both anterograde and retrograde FAT through activation of a pJNK signaling pathway, the location of pS199-phospho-tau in neurons suggests that this pathway may primarily function outside of the axon. These observations suggest that the cellular and subcellular context is important for understanding the significance of phosphorylation of specific sites on tau.

Further evidence of this is seen from the enrichment of AT8 immunoreactivity in gray matter, which is typically at low levels in adulthood, but at higher levels during development and in tauopathies (Brion et al., 1993; Watanabe et al., 1993). Despite the widespread use of the AT8 antibody to document pathological inclusions of tau, regulation of phosphorylation at this site is complex and phosphorylation at S202 and T205 are not intrinsically linked. For example, phosphorylation at both of these sites is found on tau released into the CSF. However, the level of pS202 remains constant during the course of AD, whereas pT205 levels increase (Barthélemy et al., 2020). Additionally, pS202 has been identified by mass spectroscopy as one of the most common sites of tau phosphorylation, whereas pT205 was not detected in the analysis of healthy adult rat, mouse, or human brains (Watanabe et al., 1993; Morris et al., 2015; Barthélemy et al., 2019). However, this may be due to its extremely short half-life as 50% of the pT205 signal in mouse brain lysates is lost within 60 s of death (Wang et al., 2015). Interestingly, this is consistent with the activation of PP1 by pT205 tau due to increased exposure to the PAD.

It is important to note that a fourth phosphorylation site in tau, pS208, may also be recognized by the AT8 antibody (Malia et al., 2016). pS208 has been much less studied than the other sites of this epitope, but recent mass spectrometry articles have found evidence of phosphorylation at this site in AD (Barthélemy et al., 2019; Horie et al., 2020). Soluble, but not insoluble tau purified from AD brains was phosphorylated at S208, although to a lesser extent than S202 or T205 (Horie et al., 2020). Similar to pT205, pS208 could not be identified in soluble tau from control brain tissues, but low levels were detected in control CSF (Barthélemy et al., 2019; Horie et al., 2020). While phosphorylation at this site appears to occur at low frequency, it is known to affect tau aggregation properties *in vitro* (Despres et al., 2017) and could therefore also affect tau monomer conformation in such a way as to influence the activation of downstream signaling pathways. Additional studies are needed to determine whether S208 phosphorylation of tau leads to effects on FAT.

Phosphoprotein-species are maintained by an intricate balance of kinase and phosphatase activities, which are spatially and temporally regulated. *In vitro* S199, S202, T205 all have the potential to be phosphorylated by at least seven kinases, including GSK3β and Cdk5 (Singh et al., 1995; Liu et al., 2004; Cavallini et al., 2013; Kimura et al., 2014a). Although it is unknown how many of these kinases phosphorylate tau *in vivo*, tau certainly has the potential to be a central point for multiple signaling pathways potentiating disparate downstream functions, depending on which site is modified. Equally important is switching off the signaling by dephosphorylation. The major phosphatase for tau is PP2A accounting for ~70% of the activity, but certain sites are preferentially dephosphorylated by either PP2B or PP1 (Liu et al., 2005). Interestingly, T205 is a site that is more efficiently dephosphorylated by PP1. This sets up the possibility of a negative feedback loop, an important feature of many signaling cascades.

Many questions remain about the role of tau in spatial and temporal regulation of signaling pathways, suggesting this is a rich area for further study. For example, an important feature of tau biology not addressed here relates to isoform-specific differences (Cox et al., 2016). Studies have shown that tau isoforms are phosphorylated differentially by multiple kinases and the switch from fetal to adult tau is matched by a net reduction in overall phosphorylation (Singh et al., 1996; Tuerde et al., 2018). For example, there were distinct differences between tau isoforms in the potentiation of NGF signaling with fetal 0N3R tau causing greater activation of ERK than any other isoform (Leugers et al., 2013). Additionally, we previously showed that 0N4R tau aggregates inhibited anterograde FAT to a greater extent than any other isoform (Cox et al., 2016). In this study, we used hTau40 (also known as 2N4R), the longest isoform of tau in the adult CNS as a continuation of our previous tau phosphorylation studies (Kanaan et al., 2011; Tiernan et al., 2016). This isoform is not present during development, and it has also been suggested 2N tau is preferentially sorted to the somatodendritic compartment of neurons (Zempel et al., 2017). Future studies will address whether effects on FAT elicited by pseudophosphorylation of hTau40 at AT8 sites extend to other tau isoforms. Nevertheless, recent observations suggest a much larger set of biological roles for tau than just regulation of microtubule dynamics.

## Conclusions

The data presented in this study strongly supports our hypothesis that phosphorylation of tau at individual sites can modulate tau function as a signaling hub. Tau is well known to have a highly dynamic protein structure and several lines of research suggest that phosphorylation of tau at specific sites can stabilize distinct conformations. These conformations may allow activation of diverse signaling pathways through the exposure of multiple biologically active domains such as the N-terminal PAD and others yet to be defined (Ittner et al., 2009; Leugers and Lee, 2010; Kanaan et al., 2011; Combs et al., 2016).

## Data Availability Statement

The raw data supporting the conclusions of this article will be made available by the authors, without undue reservation.

## Ethics Statement

Ethical review and approval was not required for the animal study because IACUC approval was not required for this study. All recommendations for humane use of squid set by the Marine Biological Laboratory were followed.

## Author Contributions

SM and SB conceived and designed the study, plotted and analyzed the data. M-YT expressed and purified the recombinant proteins. KB, SA, SK, and SM collected the data with additional experimental assistance from GM and SB. SM, GM, and SB wrote and edited the manuscript. All authors contributed to the article and approved the submitted version.

## Conflict of Interest

The authors declare that the research was conducted in the absence of any commercial or financial relationships that could be construed as a potential conflict of interest.
